# Effect of Iontophoresis on the Effectiveness of Nano-Hydroxyapatite and Pro-argin in In-Office Treatment of Dentin Hypersensitivity: A Split-Mouth Randomized Clinical Trial

**DOI:** 10.7759/cureus.50990

**Published:** 2023-12-23

**Authors:** Anjana Das C, Elizabeth Prabha James, Jayasree S, Parvathy V, Vidya K G, Anju Varughese, Varna Rajesh, Prasanth Balan

**Affiliations:** 1 Department of Conservative Dentistry and Endodontics, Government Dental College, Kozhikode, IND; 2 Department of Conservative Dentistry and Endodontics, Government Dental College, Thiruvananthapuram, IND; 3 Department of Conservative Dentistry and Endodontics, Amrita Vishwa Vidyapeetham, Amrita School of Dentistry, Kochi, IND

**Keywords:** nanohydroxyapatite, pro-argin, dentin sensitivity, calcium carbonate, arginine, hydroxyapatite, iontophoresis, randomized clinical trial, dentin desensitizing agents, dentin hypersensitivity

## Abstract

Background and objectives

Dentin hypersensitivity (DH) treatment is one of the main challenges dentists face in their daily clinical practice. Current therapies provide only temporary relief and require multiple applications to exhibit results, and there is a lack of evidence related to the long-term effects of these agents. Nano-hydroxyapatite (n-HA) and pro-argin (8.0% arginine-calcium carbonate) have recently been used for dentin desensitization with a one-time in-office application, but the effects are interim. However, a standard treatment protocol demands definitive or enduring results. Since iontophoresis amplifies the transport of neutral and ionized drugs across a membrane, the use of these desensitizing agents with iontophoresis may be beneficial to accomplish satisfactory results. This study was conducted to evaluate whether iontophoresis could enable better penetration of nano-hydroxyapatite and pro-argin into the dentin, enhancing and prolonging their therapeutic effect.

Materials and methods

Forty-five participants with dentin hypersensitivity in the age group of 20 to 60 years were included. In each individual, four teeth with cervical lesions (one from each quadrant) were selected and divided randomly into four groups: group I: desensitizing paste containing nano-hydroxyapatite, group II: desensitizing paste containing nano-hydroxyapatite with iontophoresis, group III: desensitizing paste containing pro-argin, and group IV: desensitizing paste containing pro-argin with iontophoresis; followed by one-time application of the agents. Sensitivity was assessed by tactile, air blast, and cold-graded thermal tests (CGTTs) before and immediately after application, after one week, and at the end of the first, third, and sixth months.

Statistical analysis

Statistical analysis was done by repeated measures ANOVA for within-group comparison. Intergroup comparison was done using one-way ANOVA and Tukey’s post-hoc test.

Results

All the groups showed a statistically significant reduction in dentin hypersensitivity (p<0.001). The reduction in hypersensitivity in various groups can be graded as group II (3.578/1.800/1.556) > group IV (3.367/1.755/1.555) > group I (2.3781/1.022/0.822) > group III (2.222/0.911/0.778) as evaluated by tactile, air blast, and cold-graded thermal tests, respectively. Group II and group IV presented a significant reduction in sensitivity levels consistent for up to six months.

Conclusion

Nano-hydroxyapatite and pro-argin can be used effectively for reducing dentin hypersensitivity. Iontophoresis can be a valuable adjunct for their improved delivery, enhancing and prolonging their effectiveness.

## Introduction

Dentin hypersensitivity is characterized by short, sharp pain arising from exposed dentin in response to stimuli typically thermal, evaporative, tactile, osmotic, or chemical, which cannot be ascribed to any other form of dental defect or pathology [1]. It is one of the most painful and least successfully treated chronic problems of the teeth. It has been reported that as many as one in every seven patients undergoing dental treatment experiences this painful condition [2].

Females are reported to have a higher incidence of dentin hypersensitivity than males. Also, the most affected patients are between 20 and 50 years of age, with a peak age group of 30 to 40 years [3]. The cervical region of the buccal surfaces of canines and premolars are the most commonly affected areas.

Dentin hypersensitivity occurs when the dentinal tubules of the exposed dentin become patent at the pulpal and outer ends. Dentinal tubules may become exposed by the loss of enamel due to abrasion, attrition, erosion, abfraction, or gingival recession and cementum loss from root surfaces.

The most widely accepted theory to explain the mechanism of pain in dentin hypersensitivity is Brännström’s hydrodynamic theory, according to which the sensitivity of dentin is the result of stimulus-induced fluid flow in the dentinal tubules and consequent nociceptor activation in the pulp-dentin border area [4].

Various treatment modalities for the management of dentin hypersensitivity focus on modifying the dentin surface or tubules by physical or chemical means, such as protein precipitation, tubular plugging, or nerve desensitization.

Dentin hypersensitivity can be managed either as an in-office treatment in the dental clinic or as a home-based application in the form of dentifrices and mouthwashes. The home-applied agents would take time to make evident results and require a considerable amount of patient compliance. Even though the in-office treatment modalities provide instantaneous pain relief, their effects are often interim. Thus, a gold standard that bestows lasting relief from dentin hypersensitivity does not exist.

Potassium is the primary agent for at-home desensitizing toothpastes that disturb the transmission of nerve endings. However, the effect of potassium nitrate is cumulative, and it may take several weeks for patients to feel any pain reduction [5]. Therapeutic agents that promote dentinal tubule occlusion are conventionally used for both in-office and at-home desensitization, the most common one being sodium fluoride (NaF). Despite being widely used, the effect of NaF varnish on dentin hypersensitivity relief is found to be limited and descending after three to six months of application. Recently, other agents using different ingredients, such as bioglasses, strontium salts, and arginine-calcium carbonate, have been developed. Pro-argin, an 8% arginine-calcium carbonate in-office desensitizing paste, is also available as a home-care toothpaste that can be recommended for use in conjunction with the in-office treatment for better results. Pro-argin forms plugs composed of arginine, calcium carbonate, and phosphate that physically seal the dentinal tubules. These plugs are found to be resistant to normal physical and acid challenges, and they instantly reduce dentin hypersensitivity by blocking tubular fluid movement. Another potent agent that can treat dentin hypersensitivity is nano-hydroxyapatite (n-HA). This is a highly biocompatible and bioactive material used for bone and tooth remineralization in medicine and dentistry, respectively. The nano-sized hydroxyapatite particles are found to possess similar morphology, ultrastructure, and crystallinity compared to tooth apatite. Previous studies have reported that nano-hydroxyapatite and pro-argin considerably reduced dentin hypersensitivity for up to four months with a single application [6].

Thus, current therapies provide only temporary relief and require multiple applications to take effect, which explains the large number of studies evaluating the reduction of pain in the short term and the lack of evidence related to the long-term effects. However, a standard treatment protocol demands much longer-lasting or permanent relief. Therefore, to improve the therapeutic effect, the application of iontophoresis along with these agents may be considered. Iontophoresis involves the use of low-ampere direct electrical current to introduce ionized or neutral drugs into tissues. It allows concentrated application of the drug to the desired area without any systemic effects. This study evaluated whether iontophoresis could enable better penetration of nano-hydroxyapatite and pro-argin into dentin, enhancing and prolonging their therapeutic effect.

## Materials and methods

This randomized clinical trial was approved by the Institutional Ethics Committee (IEC 217/2020/DCC), and the trial was registered (CTRI/2021/04/032974). It was conducted in strict adherence to the 2010 Consolidated Standards for Reporting Trials (CONSORT) statement [7]. 

Sample size calculation

In order to detect a clinically relevant difference of 20% at a 5% level of significance with 80% power of the study, the required sample size was 44 teeth for each group, which was rounded to 45 per group (a total of 180 teeth). The sample size was calculated based on a previous study by Purra et al. [8], comparing the effectiveness of two dentine desensitizers.

Participant recruitment and intervention

**Figure 1:**
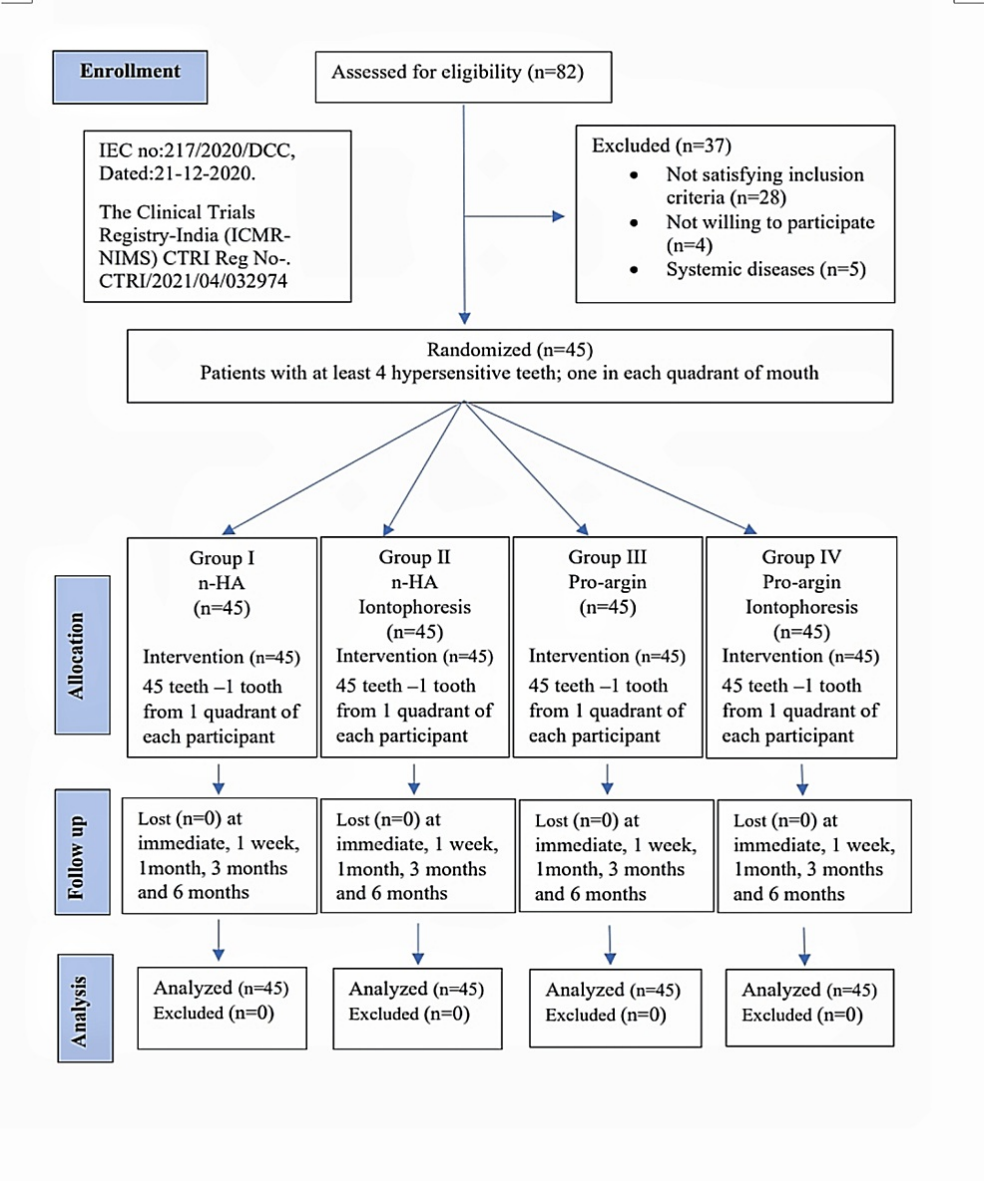
CONSORT flow diagram of the clinical trial. CONSORT: Consolidated Standards for Reporting Trials, n-HA: nano-hydroxyapatite. n: sample size (n=45 corresponds to 100% sample size).

The protocol followed the recommendations of the CONSORT statement (Figure 1). The clinical trial was carried out in a split-mouth design to reduce variability and decrease the number of participants. It was conducted on a total of 180 teeth presenting with hypersensitivity among 45 participants (four teeth in each participant).

Study population and location

The participants were selected from patients attending the Department of Conservative Dentistry and Endodontics at Government Dental College, Kozhikode, Kerala, India.

Patient screening and allocation

Eighty-two patients willing to participate in the study were screened to select those with at least four hypersensitive teeth; one in each quadrant of the mouth satisfying the inclusion criteria. Diagnosis was made based on the patient’s history, clinical examination, and pulp vitality tests. A tactile stimulus, a controlled air stimulus (evaporative stimulus), and cold water (thermal stimulus) were used to assess tooth sensitivity. 

Inclusion criteria

The participants included in the study were aged 20-60 years of both genders, having dentin hypersensitivity caused by gingival recession or cervical abrasion/erosion in the canines or premolars, with dentin loss up to 1 mm depth that did not necessitate any restorative procedures. The participants should have at least four hypersensitive teeth; one in each quadrant of the mouth and a preoperative visual analog scale (VAS) score of ≥3 but ≤ 6.

Exclusion criteria

Teeth with caries, fractures, or restorations; and those with deep periodontal pockets with probing depth >6 mm or a history of periodontal surgery within the past three months were excluded from the study. Individuals wearing orthodontic appliances or prostheses and those with any gross oral pathology or conditions like pregnancy and lactation, systemic diseases like chronic diseases, eating disorders, acute myocardial infarction within the past six months, use of a pacemaker, uncontrolled metabolic disease, and major psychiatric disorders were also excluded. Participants with a habit of smoking or alcohol abuse and those who were using desensitizing toothpaste in the previous three months were not considered in the study.

Randomization

Informed written consent was obtained from 45 participants. According to the split-mouth design, in each participant, the selected teeth were randomly divided into four groups by the lottery method using sequentially numbered, opaque, and sealed envelopes. A trained dental assistant was assigned for randomization and allocation.

Group I: Desensitizing paste containing nano-hydroxyapatite (Apagard Premio, Sangi Co. Ltd, Japan).

Group II: Desensitizing paste containing nano-hydroxyapatite with iontophoresis.

Group III: Desensitizing paste containing pro-argin (Colgate Sensitive Pro-Relief, Colgate-Palmolive India Ltd, India).

Group IV: Desensitizing paste containing pro-argin with iontophoresis.

The study design was double-blinded, i.e., both the patient and the observer were not aware of the agents used. An experienced operator was concerned with the application of the agents, and another one was assigned for the evaluation of hypersensitivity.

Evaluation of hypersensitivity

Sensitivity was assessed by tactile, air blast, and cold-graded thermal tests (CGTTs) before and immediately after the procedure, and by patient review at the end of one week, one month, three months, and six months. 

**Figure 2:**
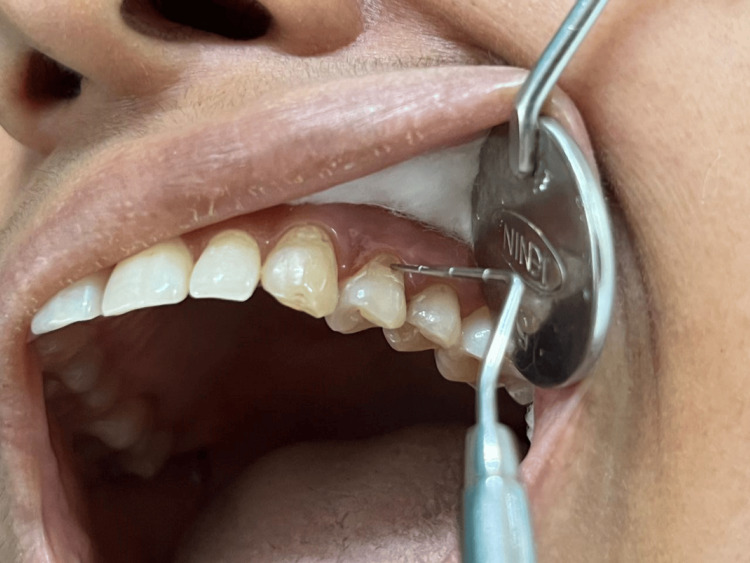
Tactile test. Tactile test on the maxillary left first premolar by a blunt probe.

In the tactile test, a blunt probe under slight manual pressure was used in a mesiodistal direction over the exposed dentin (Figure 2). The degree of hypersensitivity was scored on a 10-cm visual analog scale, which had ratings of 0-1 for no pain, 2-3 for mild pain, 4-6 for moderate pain, and 7-10 for severe pain. Subjects with baseline VAS scores ≥3 but ≤6 were accepted into the study [9].

**Figure 3:**
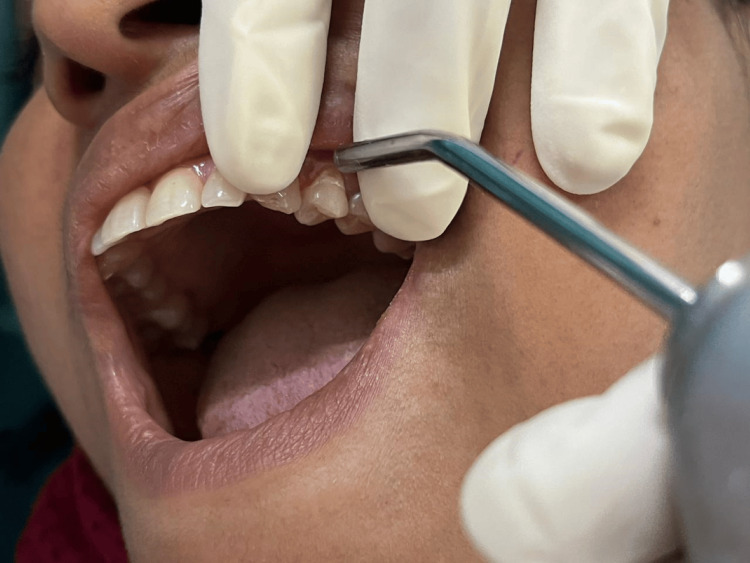
Air blast test. Air blast test on the maxillary left first premolar was done using the air component of a dental air-water syringe.

The air blast test was done by directing a blast of air (40 psi ± 5 psi) perpendicular to the hypersensitive areas of the tooth from a distance of one centimeter for 1-2 s using the air component of a dental air/water syringe. Adjacent teeth were shielded by the placement of two fingers (Figure 3). The degree of hypersensitivity was measured using Schiff’s cold air sensitivity scale, as mentioned below [10].

0-Tooth/subject does not respond to the air stimulus.

1-Tooth/subject responds to the air stimulus but does not request discontinuation of stimulus.

2-Tooth/subject responds to the air stimulus and requests discontinuation or moves from the stimulus.

3-Tooth/subject responds to the air stimulus, considers the stimulus to be painful and requests discontinuation of the stimulus.

**Figure 4:**
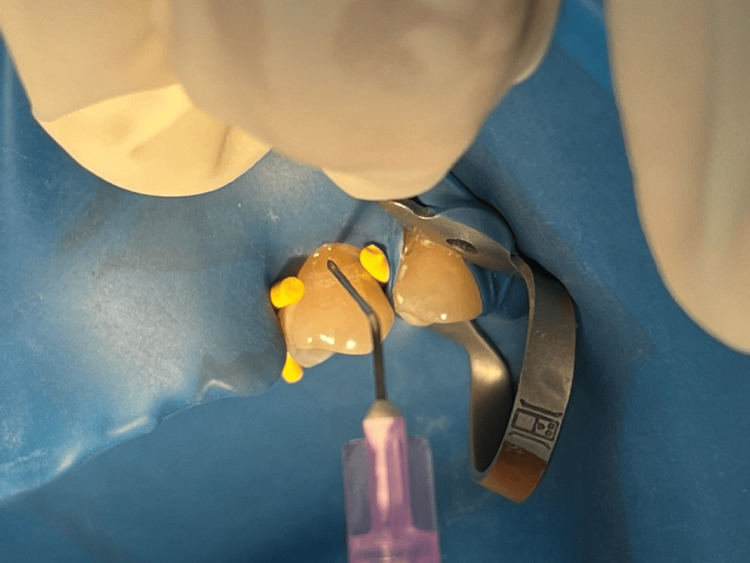
Cold-graded thermal test (CGTT). Cold-graded thermal test (CGTT) on the maxillary left first premolar by application of cold water using a syringe and needle.

The patient's response to a cold stimulus was quantitatively assessed by the cold-graded thermal test (CGTT) developed by Brough et al. [11]. It was done by serial applications of water at specific temperatures of 20°C, 10°C, and 0°C (with a variance of 1°C for each temperature interval) onto the isolated tooth surface. The desired water temperature was attained by the addition of either ice or hot water and was maintained throughout the procedure using thermal-insulated containers. The application of water over the isolated tooth surface was done by a disposable syringe until a sensitive response occurred or for a maximum of three seconds if no response occurred (Figure 4). The test was started with water at 20°C, and in case of no response, the test was repeated with a graded reduction of water temperature by 10°C up to 0°C or until a sensitive response occurred. An interval of two minutes elapsed after each application so that the tooth could return to body temperature before the next one. For data analysis, the responses of the patients to the test temperatures were converted to a ranking score as suggested by Brough et al. [11]. The ranking was assigned as follows: 4-20°C, 3-10°C, 2-0°C, and 1-insensitive to the lowest test temperature [11].

The brushing habits of the participants were evaluated through a questionnaire that included the brushing method, technique, frequency, and agent used (Appendix 1, Figure 12).

Method of application

**Figure 5:**
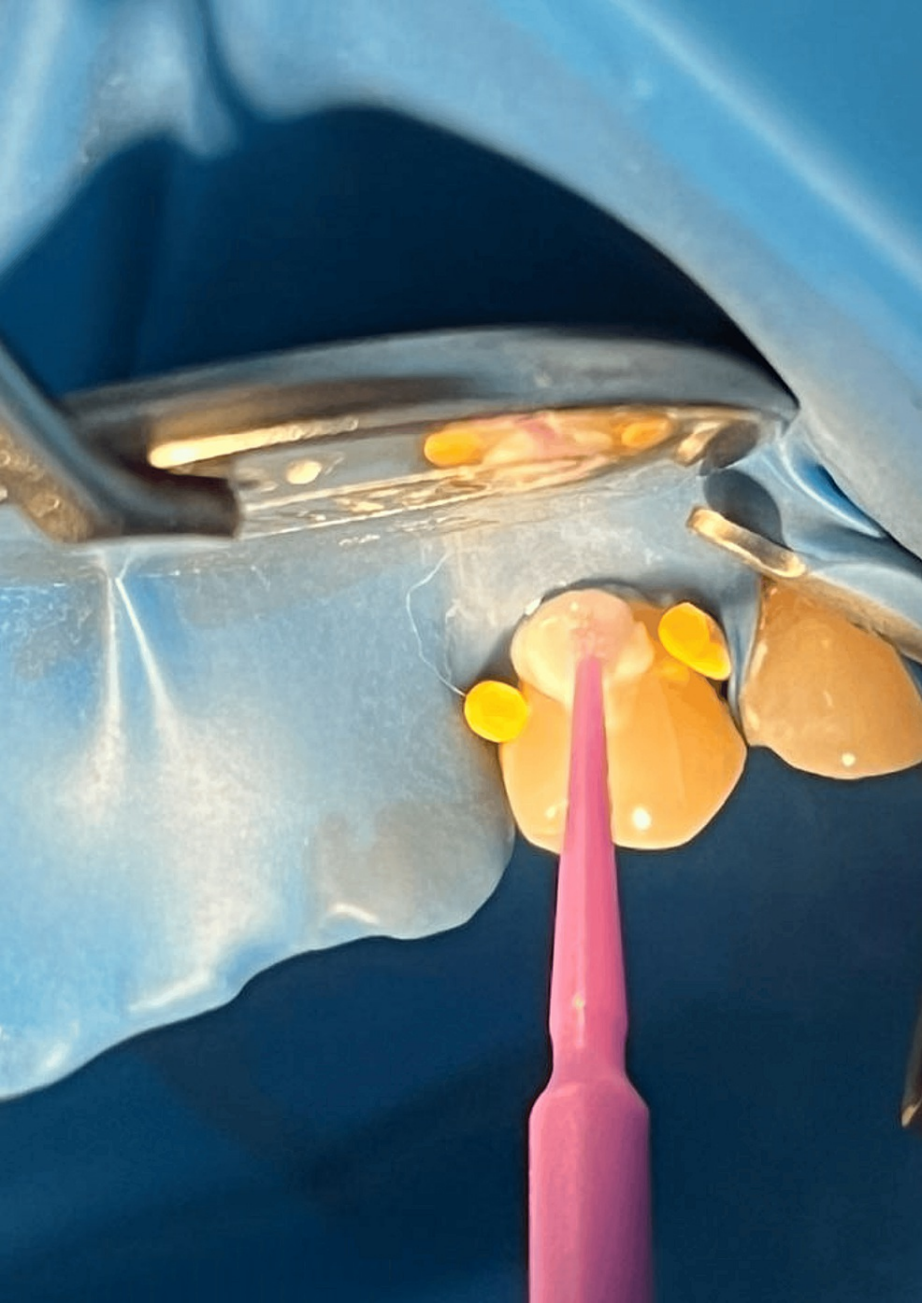
Application of desensitizing agent. Desensitizing paste was applied on the maxillary left first premolar using a microapplicator.

The mode of application followed the guidelines [6,12], which are briefly described. For groups I and III, 0.25 g of the assigned desensitizing paste was applied to the isolated area with hypersensitive lesions using disposable microapplicators for two minutes (Figure 5). A rotary cup in a contra-angled handpiece at moderate to high speed was used to blend and polish the paste at its site of application for approximately 60 seconds.

**Figure 6:**
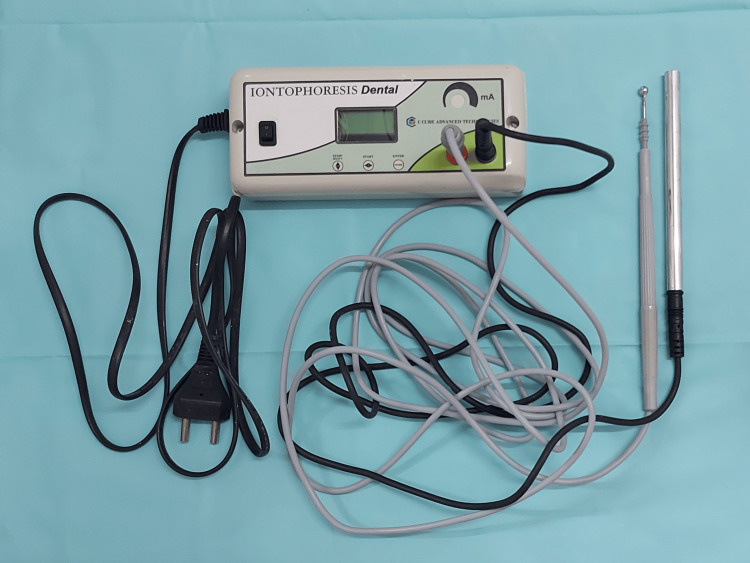
Dental iontophoresis unit. Dental iontophoresis unit consisting of two electrodes (patient electrode and treatment electrode).

**Figure 7:**
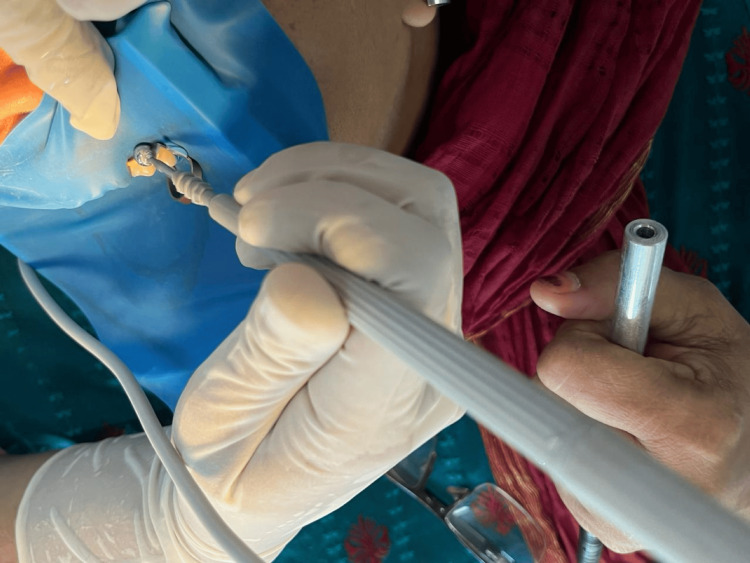
Application of iontophoresis. Iontophoretic probe was placed on the maxillary left second premolar after the application of desensitizing paste.

For groups II and IV, in addition to the above procedure, an iontophoretic unit (Dental Iontophoresis, C Cube Advanced Technologies, Bangalore, India) was used to deliver current (Figures 6, 7). A current of 2.5 mA, 15 V, was delivered till the patient felt a tingling sensation, or for a maximum of two minutes. This was repeated three times during a single appointment at an interval of two minutes between each application. For groups I and III, as a part of blinding, the iontophoretic probe was applied in the same manner as mentioned above but without passing current.

The participants were instructed to avoid eating or drinking for two hours and brushing for 12 hours after the procedure. The proper brushing technique was demonstrated after the procedure. After oral hygiene instructions and diet counseling, participants were advised to use regular toothpaste without any desensitizing agents.

Outcome measurement

The clinical evaluation of post-treatment sensitivity scores was done immediately after treatment, and during patient review on the seventh day, and at the end of the first, third, and sixth months. The assessment of hypersensitivity at various time intervals was executed and recorded by the same evaluator, following the same methodology employed at the baseline examinations.

Statistical analysis

The collected data were subjected to statistical analysis using Statistical Software SPSS Version 20 (IBM Corp., Armonk, New York, USA) with a significance level set at p≤0.05. To obtain within-group comparisons, mean values and standard deviations at different time periods were analyzed by repeated measures ANOVA. Intergroup comparison was done using a one-way ANOVA and Tukey’s post-hoc test. The operator and the outcome assessor were calibrated, and the kappa statistical value was noted to be 0.75 and 0.78, respectively.

## Results

Eighty-two patients were clinically screened, and those who were either ineligible or absent on the day of the dental treatment were excluded. Subsequently, 45 adult participants were recruited, which included 18 males and 27 females (Figure 1). The data were collected, tabulated, and statistically analyzed using the Statistical Software SPSS Version 20. The results were considered statistically significant at p≤0.05. All the groups showed a statistically significant reduction in dentin hypersensitivity (p<0.001) (Table 1).

**Table 1:** Comparison of mean scores at baseline and after six months using ANOVA. CGTT: cold-graded thermal test; p: level of significance. *p≤0.05 was considered statistically significant.

Groups	Evaluations	Baseline	Six months	Mean difference	F-statistic	p-value
Group I	Tactile test	4.4667	2.2889	2.3781	52.38	<0.001*
	Air blast test	2.222	1.200	1.022		
	CGTT	2.088	1.266	0.822		
Group II	Tactile test	4.400	0.822	3.578	153.02	<0.001*
	Air blast test	2.222	0.422	1.800		
	CGTT	2.022	0.466	1.556		
Group III	Tactile test	4.444	2.222	2.222	91.86	<0.001*
	Air blast test	2.177	1.2660.	0.911		
	CGTT	2.022	1.244	0.778		
Group IV	Tactile test	4.311	0.844	3.367	134.79	<0.001*
	Air blast test	2.177	0.422	1.755		
	CGTT	1.977	0.422	1.555		

**Figure 8:**
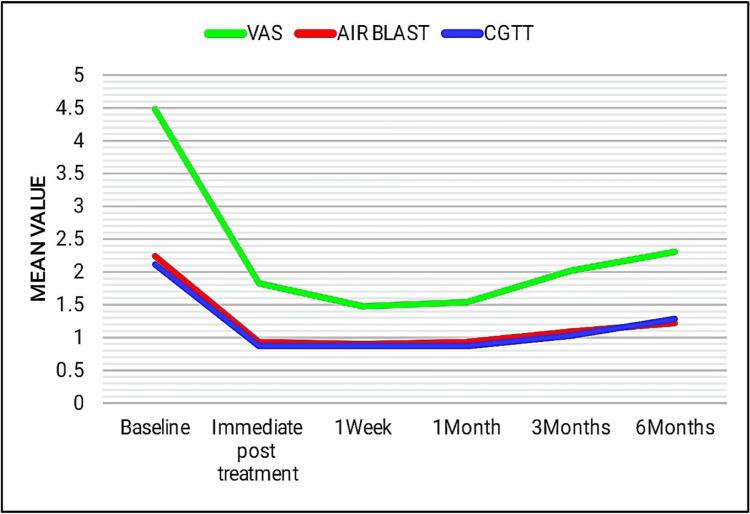
Line diagram showing the comparison of mean scores of group I in tactile (VAS score), air blast, and cold-graded thermal tests at different time intervals. VAS: visual analog scale (tactile test) and CGTT: cold-graded thermal test.

**Figure 9:**
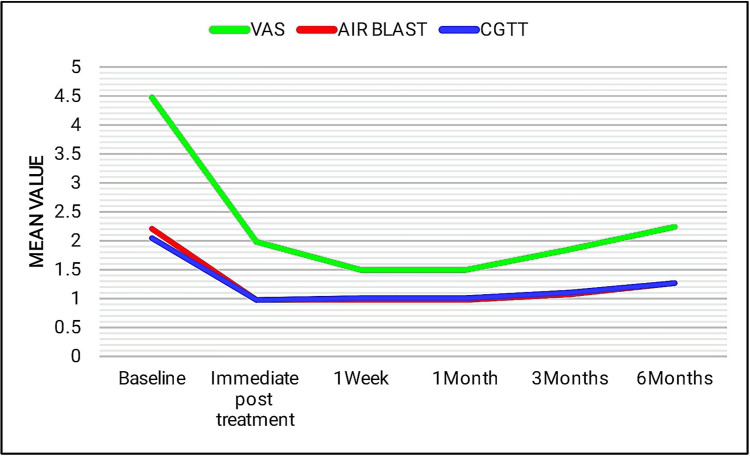
Line diagram showing the comparison of mean scores of group III in tactile (VAS score), air blast, and cold-graded thermal tests at different time intervals. VAS: visual analog scale (tactile test) and CGTT: cold-graded thermal test.

Even though group I and group III showed a statistically significant reduction, at the end of one month there was an increase in the sensitivity scores to all three stimulus tests (Figures 8, 9).

**Figure 10:**
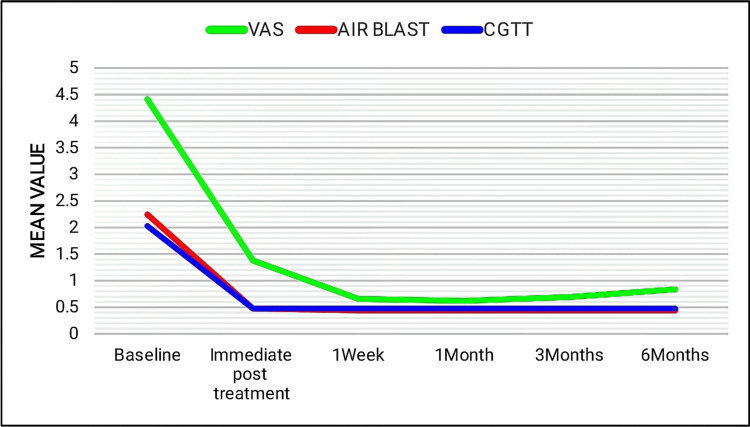
Line diagram showing the comparison of mean scores of group II in tactile (VAS score), air blast, and cold-graded thermal tests at different time intervals. VAS: visual analog scale (tactile test) and CGTT: cold-graded thermal test.

**Figure 11:**
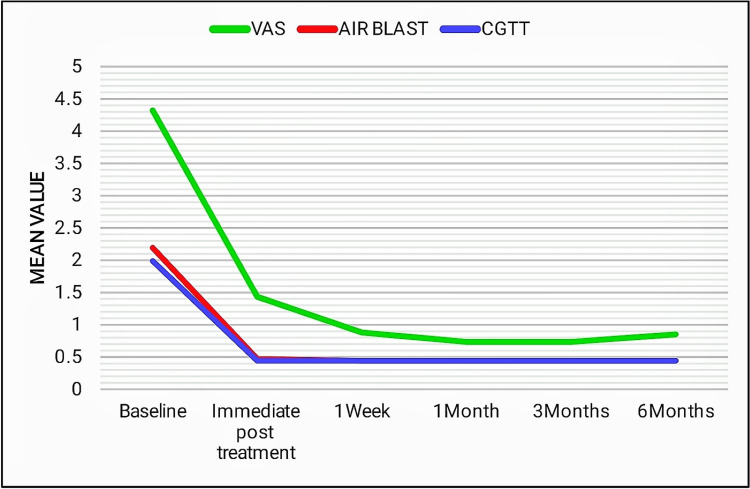
Line diagram showing the comparison of mean scores of group IV in tactile (VAS score), air blast, and cold-graded thermal tests at different time intervals. VAS: visual analog scale (tactile test) and CGTT: cold-graded thermal test.

**Figure 12:**
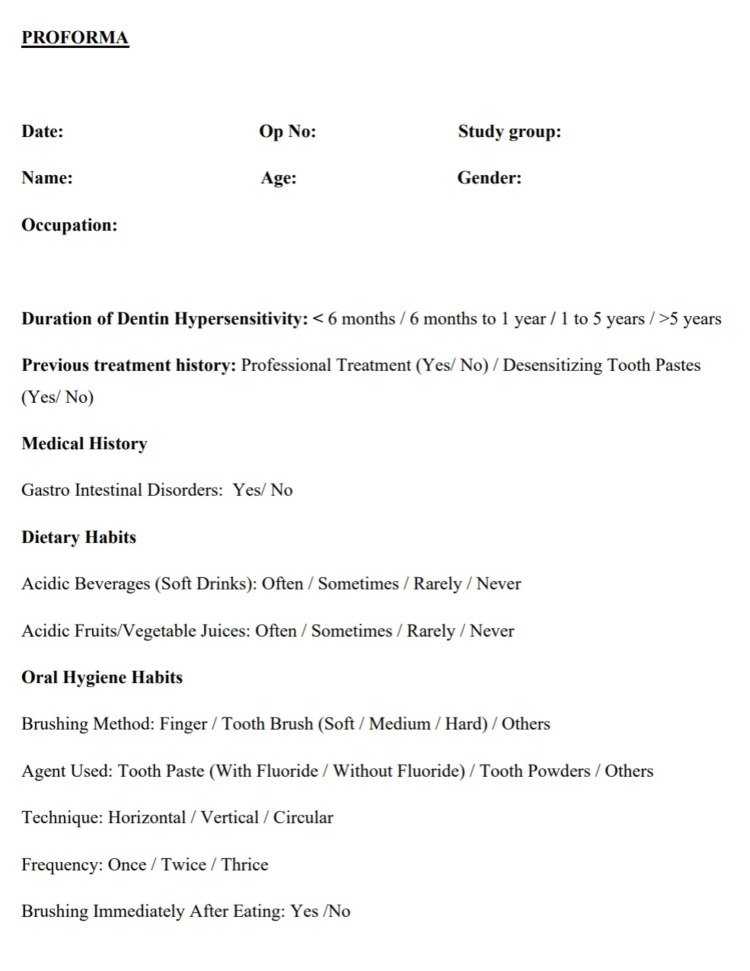
Participant questionnaire.

Group II and group IV presented with a significant reduction in sensitivity levels, which was consistent for up to six months with respect to air blast and cold-graded thermal tests. To tactile stimulus, group IV exhibited a slight increase in the sensitivity scores after three months, whereas group II showed a slight increase at the end of one month (Figures 10, 11).

Analysis between groups by one-way ANOVA showed that group II exhibited the greatest reduction in dentin hypersensitivity in all the tested parameters, followed by group IV, group I, and then group III at all intervals (Table 2).

**Table 2:** Comparison of mean scores between groups at different time intervals (one-way ANOVA). SD: standard deviation, N: sample size, p: level of significance. *p≤0.05 was considered statistically significant.

Evaluations	Test groups	N (%)	Tactile test			Air blast test			Cold-graded thermal test		
			Mean	SD	p-value	Mean	SD	p-value	Mean	SD	p-value
Baseline	Group I	45 (100%)	4.4667	1.15994	0.904	2.2222	0.79455	0.968	2.0889	0.79264	0.926
	Group II	45 (100%)	4.4000	1.13618		2.2222	0.79455		2.0222	0.78303	
	Group III	45 (100%)	4.4444	0.94281		2.1778	0.77720		2.0222	0.78303	
	Group IV	45 (100%)	4.3111	0.94922		2.1778	0.77720		1.9778	0.75344	
Immediate	Group I	45 (100%)	1.8222	0.96032	0.006*	0.9111	0.70137	<0.001*	0.8444	0.60135	<0.001*
	Group II	45 (100%)	1.3778	0.83364		0.4444	0.50252		0.4222	0.50452	
	Group III	45 (100%)	1.9778	1.03328		0.9556	0.63802		0.9556	0.63802	
	Group IV	45 (100%)	1.4222	0.98832		0.4667	0.50452		0.4667	0.49949	
One week	Group I	45 (100%)	1.4667	0.99087	<0.001*	0.8889	0.68165	<0.001*	0.8444	0.56228	<0.001*
	Group II	45 (100%)	0.6444	0.67942		0.4222	0.49949		0.4222	0.50452	
	Group III	45 (100%)	1.4889	1.01404		0.9556	0.63802		0.9778	0.65674	
	Group IV	45 (100%)	0.8667	0.84208		0.4222	0.49949		0.4667	0.49949	
One month	Group I	45 (100%)	1.5333	0.96766	<0.001*	0.9111	0.66818	<0.001*	0.8667	0.54772	<0.001*
	Group II	45 (100%)	0.6000	0.61791		0.4222	0.49949		0.4222	0.50452	
	Group III	45 (100%)	1.4889	1.05792		0.9556	0.63802		1.0000	0.67420	
	Group IV	45 (100%)	0.7111	0.81526		0.4222	0.49949		0.4667	0.49949	
Three months	Group I	45 (100%)	2.0000	1.06600	<0.001*	1.0667	0.71985	<0.001*	1.0000	0.60302	< 0.001*
	Group II	45 (100%)	0.6889	0.59628		0.4222	0.49949		0.4222	0.50452	
	Group III	45 (100%)	1.8444	1.04350		1.0444	0.67270		1.0889	0.66818	
	Group IV	45 (100%)	0.7111	0.81526		0.4222	0.49949		0.4667	0.49949	
Six months	Group I	45 (100%)	2.2889	1.10005	<0.001*	1.2000	0.69413	<0.001*	1.2667	0.65366	<0.001*
	Group II	45 (100%)	0.8222	0.71633		0.4222	0.49949		0.4222	0.50452	
	Group III	45 (100%)	2.2222	0.99747		1.2667	0.71985		1.2444	0.67942	
	Group IV	45 (100%)	0.8444	0.76739		0.4222	0.49949		0.4667	0.49949	

However, Tukey’s post-hoc analysis showed no statistically significant difference between group II and group IV (Table 3).

**Table 3:** Multiple comparisons of mean scores at six months of review using Tukey’s HSD test. CGTT: cold-graded thermal test, HSD: honestly significant difference, p: level of significance. *The mean difference is significant at p-value≤0.05.

Evaluations	(I) Group	(J) Group	Mean difference (I-J)	p-value	95% Confidence interval	
					Lower bound	Upper bound
Tactile test	Group II	Group I	−1.46667^*^	0.000	−1.9639	−0.9695
		Group III	−1.40000^*^	0.000	−1.8972	−0.9028
		Group IV	−0.02222	0.999	−0.5194	0.4750
	Group IV	Group I	−1.44444^*^	0.000	−1.9416	−0.9472
		Group II	0.02222	0.999	−0.4750	0.5194
		Group III	−1.37778^*^	0.000	−1.8750	−0.8806
Air blast test (evaporative stimuli)	Group II	Group I	−0.77778^*^	0.000	−1.1125	−0.4430
		Group III	−0.84444^*^	0.000	−1.1792	−0.5097
		Group IV	0.00000	1.000	−0.3347	0.3347
	Group IV	Group I	−0.77778^*^	0.000	−1.1125	−0.4430
		Group II	0.00000	1.000	−0.3347	0.3347
		Group III	−0.84444^*^	0.000	−1.1792	−0.5097
CGTT (thermal stimuli)	Group II	Group I	−0.80000^*^	0.000	−1.1227	−0.4773
		Group III	−0.77778^*^	0.000	−1.1005	−0.4551
		Group IV	0.04444	0.984	−0.2782	0.3671
	Group IV	Group I	−0.84444^*^	0.000	−1.1671	−0.5218
		Group II	−0.04444	0.984	−0.3671	0.2782
		Group III	−0.82222^*^	0.000	−1.1449	−0.4995

When desensitizing pastes were used along with iontophoresis, the reduction in sensitivity was persistent for up to six months.

## Discussion

This study has evaluated the effect of iontophoresis on the effectiveness of desensitizing agents like nano-hydroxyapatite and pro-argin. The clinical trial was carried out in a double-blinded design to avoid bias. A split-mouth design, as suggested by Torres et al. [13] and Pradeepkumar et al. [14], was adopted to facilitate the evaluation of all the groups under the same oral conditions like dietary and oral hygiene habits, pain perception, and psychosomatic factors. This can minimize inter-participant variability and requires fewer participants than a similar parallel-group trial with the same power.

Evaluation of hypersensitivity plays a crucial role in the assessment of desensitizing agents. The use of more than one stimulus test was recommended by Ide et al. [15] because subjective reproducibility would be less in a single test. According to the guidelines for the design and conduct of clinical trials on dentin hypersensitivity by Holland et al. [1], dentin hypersensitivity may differ for different stimuli; therefore, at least two hydrodynamic stimuli should be used, and the least severe stimulus should be used first. In the present study, the tactile test was done first, followed by an air blast and then cold-graded thermal tests. Three stimulus tests were used in the studies by Gopinath et al. [16], Brough et al. [11], and Brahmbhatt et al. [17]. Each of the three stimulus tests was performed at an interval of five minutes to minimize interactions between the stimuli, as suggested by Pradeep et al. [18] and Praveen et al. [19].

Nano-hydroxyapatite-containing toothpaste exhibited a significant reduction in sensitivity in the present study, which was in accordance with the studies by Wang et al. [20], Jena et al. [6], and Gopinath et al. [16]. The nano-hydroxyapatite may act as a reservoir of calcium and phosphate ions, maintaining a supersaturation of these ions with respect to the tooth apatite, thus causing remineralization by mineral deposition on the tooth surface. It has been reported that nano-hydroxyapatite can directly fill up the micropores on tooth surfaces, where it acts as a template, causing apatite deposition by attracting calcium and phosphate ions from the surrounding environment (saliva, mouth rinses, dentifrices, etc.) to the tooth tissue, thereby promoting apatite crystal integrity and growth [21].

The pro-argin paste contains arginine (a positively charged amino acid), which is buffered to a physiological pH with bicarbonate and calcium carbonate. Studies by Porto et al. [22] and Sauro et al. [23] have shown that the plugs formed within the dentin tubules were composed of arginine, calcium carbonate, and phosphate. This plug minimized sensitivity effectively by reducing the dentinal fluid flow. In the present study, a single application of pro-argin paste resulted in a reduction of sensitivity immediately after application; and at one week, one month, three months, and six months recall. The reduction in mean air blast and CGTT scores was persistent throughout the six months, whereas tactile scores showed the greatest reduction at a one-week and one-month review, after which the values increased but were still significantly different from the baseline values. This was consistent with the studies by Schiff et al. [24] and Kar et al. [25], in which a significant reduction of sensitivity was maintained for a period of four weeks after a single application. However, the results contradict the findings by Torres et al. [13], where a single application of the pro-argin-containing paste provided instant relief from sensitivity, but the scores increased to baseline values at the third-week follow-up. A study by Wang et al. [20] reported a significantly increasing effect of pro-argin through a three-month period, contrary to the result of the present study. This increasing effect might have occurred because they studied the in-office application along with the at-home application of the same for three months.

Desensitization by iontophoresis has been explained by different hypotheses, such as the formation of reparative dentin, induction of paresthesia by altering the sensory mechanism of pain conduction, and microprecipitation of calcium fluoride across the dentinal tubules, thus blocking the hydrodynamically mediated stimuli that induce pain. Iontophoresis amplifies the transport of both ionized and neutral drugs across a membrane by the application of electric current. Iontophoretic drug delivery takes place by electrophoresis, electro-osmosis, and electropermeabilization. Electrophoresis improves the transfer of ionized drugs, whereas electro-osmosis intensifies the transport of both neutral and ionized drugs.

Electropermeabilization increases the intrinsic permeability of the membrane and alters the permeation pathways across the membrane. Brahmbhatt et al. [17] observed that the desensitization effect was prolonged with fluoride iontophoresis than topical fluoride application alone. In this study, iontophoresis enhanced and prolonged the desensitizing effects of both nano-hydroxyapatite and pro-argin. Participants treated with pro-argin and iontophoresis showed a considerable reduction in sensitivity scores to tactile tests until the third month, but there was a slight increase in the scores in the sixth month.

A slight increase in sensitivity seen at the third and sixth-month review in our study could be explained by the findings of the study by Petrou et al. [26], according to which the movement of dentinal fluid within the tubules was not completely inhibited even though there was obliteration of the dentinal tubules.

Limitations of the study

The sensitivity scores at each stage were determined by converting the subjective feedback into objective data. Thus, the results were dependent upon the patient’s interpretation, which considerably reduced the reliability of the scoring. Another main limitation is the short duration of the study and the small sample size. Patients with periodontitis may provide false positive responses of hypersensitivity, even if the pocket depth is less than 6 mm, which may interfere with the results of the study. In this study, we did not come across patients suffering from periodontitis, probably because the participants were recruited from patients who attended the Department of Conservative Dentistry. The majority of the patients were in the age group of 20 to 40 years with a normal probing depth of the gingival sulcus. In order to obtain enduring results with the treatment, emphasis should be given to the elimination of underlying causative factors. The longevity of the plugs obliterating the tubule lumen is still under question and should be tested in the future using methods such as scanning electron microscopy and hydraulic conductivity tests.

## Conclusions

Within the limits of the present study, it can be concluded that both nano-hydroxyapatite and pro-argin can be used effectively for reducing dentin hypersensitivity. Iontophoresis can be a valuable adjunct for improved delivery of nano-hydroxyapatite and pro-argin, thereby enhancing and prolonging their desensitizing effects. However, long-term studies with larger sample sizes are required for more conclusive results. Further research is needed for the development of better and more precise diagnostic tests and therapeutic agents, and to assess the long-term effects of the existing therapeutic procedures, so as to lay out guidelines for use in routine clinical practice. The evolution of a therapy that can provide instant and lasting relief would be of great assistance to clinicians in treating dentin hypersensitivity.

## Appendices

Appendix 1 
